# Elucidating the Mechanism of *Weissella*-dependent Lifespan Extension in *Caenorhabditis elegans*

**DOI:** 10.1038/srep17128

**Published:** 2015-11-25

**Authors:** Jiyun Lee, Gayeung Kwon, Young-Hee Lim

**Affiliations:** 1 Department of Public Health Science (Brain Korea 21 PLUS program), Graduate School, Korea University, Seoul 136-701, Republic of Korea; 2 School of Biosystem and Biomedical Science, College of Health Science, Korea University, Seoul, Republic of Korea; 3 Department of Laboratory Medicine, Korea University Guro Hospital, Seoul, Republic of Korea

## Abstract

The mechanism whereby lactic acid bacteria extend the lifespan of *Caenorhabditis elegans* has previously been elucidated. However, the role of *Weissella* species has yet not been studied. We show that *Weissella koreensis* and *Weissella cibaria* significantly (*p* < 0.05) extend the lifespan of *C. elegans* compared with *Escherichia coli* OP50 and induce the expression of several genes related to lifespan extension (*daf-16, aak-2, jnk-1, sod-3* and *hif-1*). Oral administration of *Weissella* altered reactive oxygen species (ROS) production and lowered the accumulation of lipofuscin and increased locomotor activity (which translates to a delay in ageing). Moreover, *Weissella*-fed *C. elegans* had decreased body sizes, brood sizes, ATP levels and pharyngeal pumping rates compared with *E. coli* OP50-fed worms. Furthermore, mutations in *sod-3*, *hif-1* or *skn-1* did not alter lifespan extension compared with wild-type *C. elegans*. However, *C. elegans* failed to display lifespan extension in loss-of-function mutants of *daf-16*, *aak-2* and *jnk-1*, which highlights the potential role of these genes in *Weissella*-induced longevity in *C. elegans*. *Weissella* species extend *C. elegans* lifespan by activating DAF-16 via the c-Jun N-terminal kinase (JNK) pathway, which is related to stress response, and the AMP-activated protein kinase (AMPK)-pathway that is activated by dietary restriction.

*Weissella* species are lactic acid bacteria that have only recently been classified as a new genus. *Weissella* species are found in fermented foods, including Korean traditional fermented vegetables and kimchi, sugar cane and the intestinal tracts of humans and other animals^1^. Fermented foods, including kimchi, possess diverse lactic acid bacteria, the composition of which effects fermentation and the sensory properties of kimchi^2^. Kimchi is a well-known probiotic food, with similar health benefits to probiotic yogurt. Additionally, kimchi has a range of other health benefits including the promotion of brain-, skin- and colorectal-health as well as strengthening the immune system; kimchi has been shown to be effective against cancer, obesity, constipation and high cholesterol; it also has fibrolytic, antioxidative and antiageing properties^3^. Recently, *Weissella* species were identified as one of the main fermenters in kimchi^2^. *Weissella* species are more resistant to acidic and anaerobic conditions compared with *Leuconostoc* species^4^. *Weissella* also have unusual interpeptide bridges in the peptidoglycan layer that distinguish these bacteria from other lactobacilli^5^. However, in contrast to other lactic acid bacteria, the possible beneficial effects of *Weissella* spp. on humans require further study.

*Caenorhabditis elegans* is a small, free-living soil nematode used in various fields of research. *C. elegans* is a particularly useful model to study ageing because of its short lifespan and the fact that it is amenable to genetic analyses^6^. Providing lactic acid bacteria as a food source instead of *E. coli* OP50 increases the average lifespan of *C. elegans*^7^,^8^,^9^. Several studies have described the mechanisms whereby lactic acid bacteria extend the lifespan of *C. elegans*, but the role of *Weissella* species remains unknown. *Lactobacillus rhamnosus* extends the lifespan of *C. elegans* by modulating the DAF-2/DAF-16 signalling pathway^10^ and facilitates resistance to oxidative stress in *C. elegans*, as demonstrated by the increased survival of *C. elegans* upon H_2_O_2_-induced stress. *Bifidobacterium infantis* prolongs the lifespan of *C. elegans* through activation of *skn-1* (which is regulated by the p38 MAPK pathway) in a dose-dependent manner; this effect was not induced by dietary restriction, which means that *B. infantis* did not promote longevity through the activation of the host defence system via DAF-16^9^. Dietary restriction has been shown to extend the lifespan of animals, including humans^11^; however, this remains controversial^12^. It is not clear whether lactic acid bacteria induce dietary restriction, thereby extending the lifespan of *C. elegans*. Dietary restriction can regulate the lifespan of *C. elegans* via the insulin/IGF-1 signalling (IIS) and target of rapamycin (TOR) pathways^13^. These pathways (and others) induce the DAF-16/FOXO transcription factor^14^, which in turn regulates various genes involved in regulating longevity, stress response, metabolism and development. DAF-16/FOXO is therefore indispensable in stress resistance as well as in lifespan regulation^15^. Moreover, JNK-1 is associated with stress response in vertebrates and is a positive regulator of DAF-16. In addition, AAK-2, the *C. elegans* homologue of AMPK, is involved in DAF-16/FOXO activation and promotes longevity during periods of glucose restriction^16^.

In this study, we investigated whether *Weissella* species extend the lifespan of *C. elegans*. We also conducted an attraction assay for *C. elegans* towards *Weissella* species and *E. coli* OP50. To elucidate the mechanism underlying *Weissella*-mediated lifespan extension in *C. elegans*, the lifespan of worms was measured using loss-of-function mutants.

## Results

### *Weissella* promotes *C. elegans* longevity

**Figure 1:**
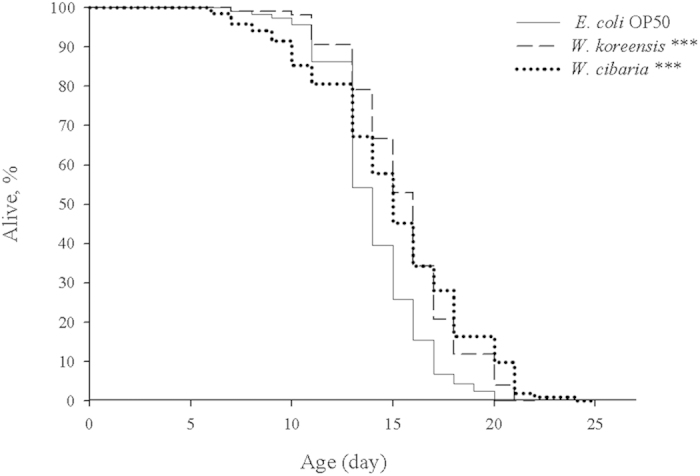
The Effect of *Weissella* on the lifespan of *C. elegans* (N2). After being fed on an *E. coli* OP50 lawn for 3 days, young adult worms were transferred to a fresh mNGM plate lawned with *E. coli* OP50, *W. koreensis* or *W. cibaria*; ^***^p < 0.001.

Feeding nematodes on a lawn of *W. koreensis* or *W. cibaria* significantly (*p* < 0.001) increased the mean lifespan (MLS) of worms compared with the group fed on the *E. coli* OP50 lawn (Table 1). *W. koreensis* was more effective than *W. cibaria* in increasing the MLS of worms. The survival rates of the worms were higher in both *W. cibaria-*fed and *W. koreensis*-fed worms compared with *E. coli* OP50-fed worms after 13 days (Fig. 1). The complete lifespan data of wild-type and mutant *C. elegans* are provided in Table S1.

### ROS production

To determine the effect of *Weissella* on ROS production in *C. elegans*, total ROS levels of worms fed on *E. coli* OP50 or *Weissella* were measured. The two *Weissella* species showed opposing effects regarding ROS production (Fig. S1), with ROS levels 43.7% lower and 37.5% higher in *W. koreensis*-fed and *W. cibaria*-fed *C. elegans,* respectively, compared with *C. elegans* fed *E. coli* OP50.

### The effects of *Weissella* on age-related biomarkers in *C. elegans*

**Figure 2:**
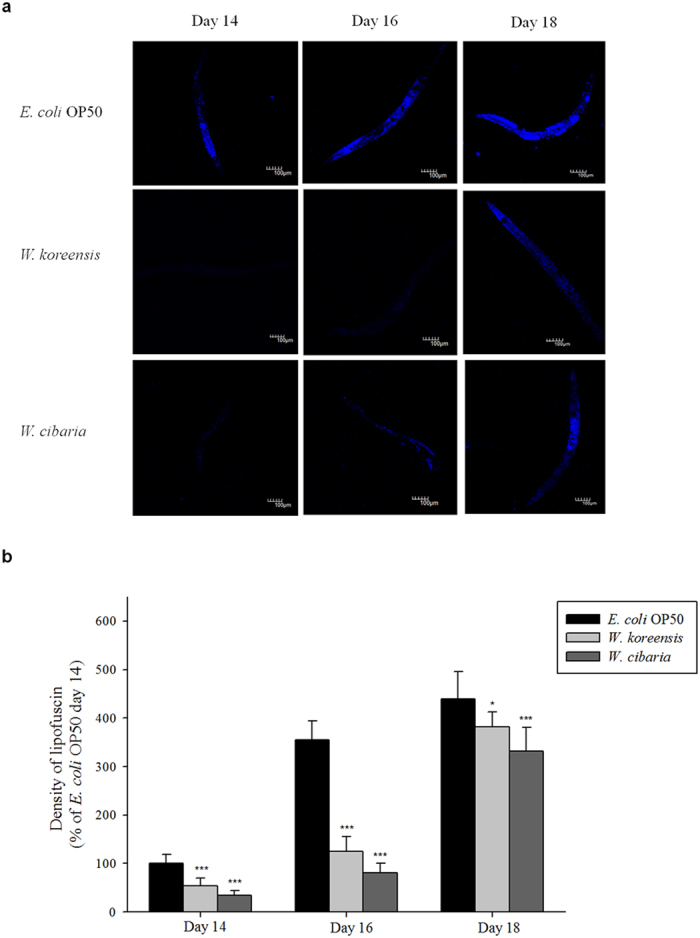
Lipofuscin accumulation in *C. elegans* (N2) fed *E. coli* OP50, *W. koreensis* and *W. cibaria.* (**a**) Fluorescence of lipofuscin in worms fed *E. coli* OP50, *W. koreensis* and *W. cibaria* on days 14, 16 and 18. Scale bar = 100 μm. (**b**) Fluorescence was quantified using ImageJ software. The graph depicts the mean percentage in arbitrary units relative to that of control worms fed *E. coli* OP50 on day 14. Ten worms were used for each measurement; ^**^p < 0.01, ^***^p < 0.001.

Age-related changes in *C. elegans* include changes in body movement, pharyngeal pumping rate and body size. Lipofuscin accumulation (a biomarker of ageing) can be determined by autofluorescence, but there is considerable inter-individual variation in lipofuscin levels in age-matched *C. elegans*^17^. We found that autofluorescence significantly decreased in *Weissella*-fed worms compared with *E. coli* OP50-fed worms (Fig. 2). Although lipofuscin fluorescence was significantly higher on day 14 in the *E. coli* OP50-fed group compared with either of the *Weissella*-fed groups, none of the three groups showed large volumes of lipofuscin. By day 16, lipofuscin levels in the *W. koreensis* and *W. cibaria* groups were 35.1% and 22.9%, respectively, of the level observed in the *E. coli* OP50 group. After 18 days, lipofuscin levels in the *W. koreensis* and *W. cibaria* groups increased to 86.9% and 75.3%, respectively, of that observed in *E. coli* OP50.

**Figure 3:**
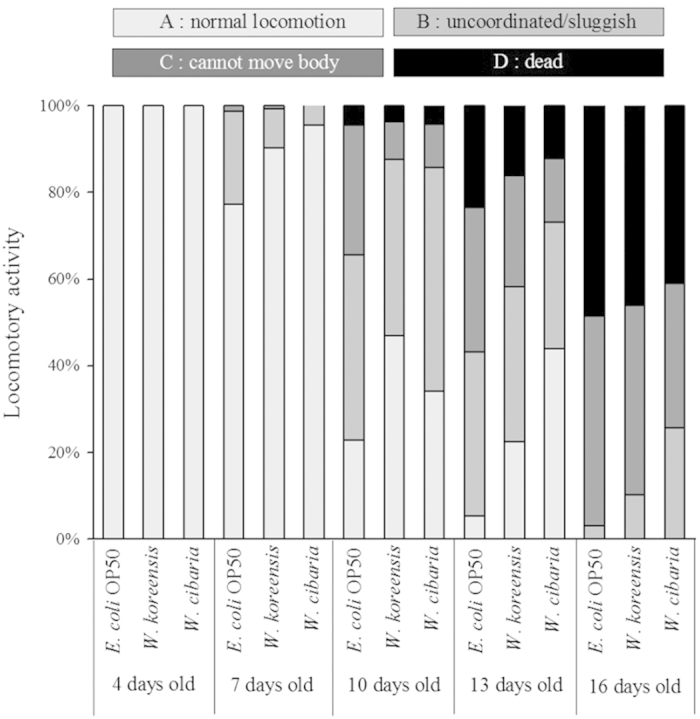
The locomotor activity of *C. elegans* (N2) fed *E. coli* OP50 or *Weissella*. L4-stage worms fed *E. coli* OP50 for 3 days after hatching were transferred to fresh mNGM plates with 20 mg of *E. coli* OP50 or *Weissella* lawn. Nematodes were classified into four classes based on their locomotion: class (**A**) normal coordinated sinusoidal locomotion; class (**B**) uncoordinated and/or sluggish movement; class (**C**) no movement except head or tail in response to prodding; and class (**D**) dead worms. The bars indicate the proportion of each class at the designated time.

**Figure 4:**
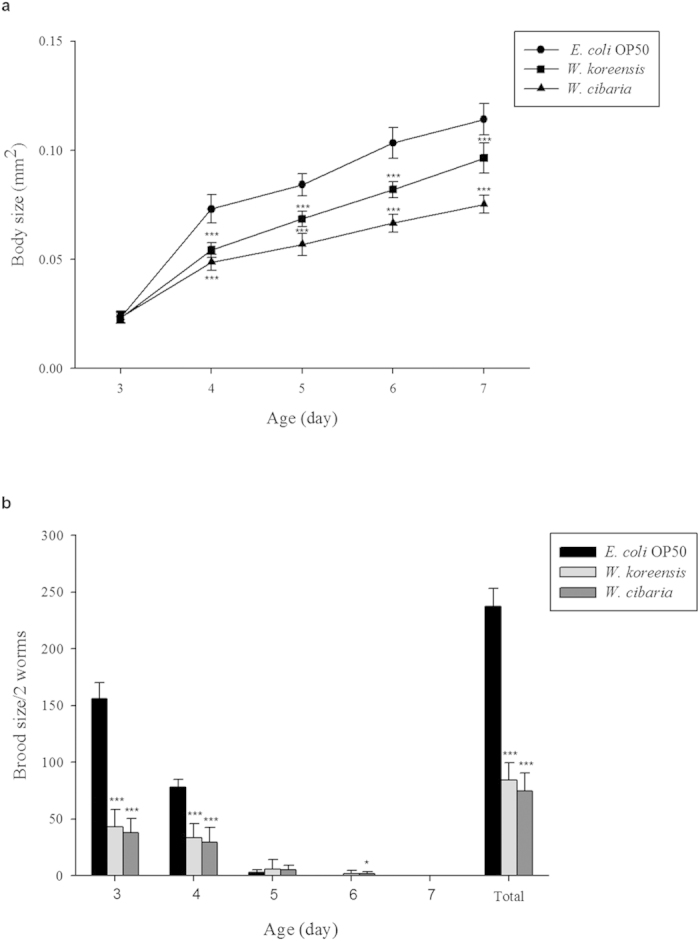
Influence of *Weissella* on *C. elegans* (N2) body size and brood size. (**a**) L4-stage *C. elegans* were transferred to fresh mNGM plates seeded with each bacterial species on day 3, and body size was determined from 20 worms for each bacterial species. (**b**) Total brood size was determined from 30 animals, and values represent the mean for each pair of worms. Significant differences shown are relative to *E. coli* OP50 (^*^p < 0.05, ^***^p < 0.001).

We measured the locomotory rate of *C. elegans* on days 4, 7, 10, 13 and 16 and found that the proportion of worms displaying sinusoidal locomotion (class A) was higher in *Weissella-*fed worms compared with worms fed *E. coli* OP50 (Fig. 3). Body size increased with age in all groups; however, the *Weissella*-fed worms were smaller than *E. coli*-fed worms (Fig. 4a). Meanwhile, brood size was smaller in *Weissella*-fed worms compared with the control group (Fig. 4b). Interestingly, the reproductive periods in the *Weissella*-fed groups were slightly longer compared with the *E. coli* OP50-fed group. The mean total brood sizes for each pair of worms were 237, 84 and 74 in worms fed *E. coli* OP50, *W. koreensis* and *W. cibaria*, respectively.

**Figure 5:**
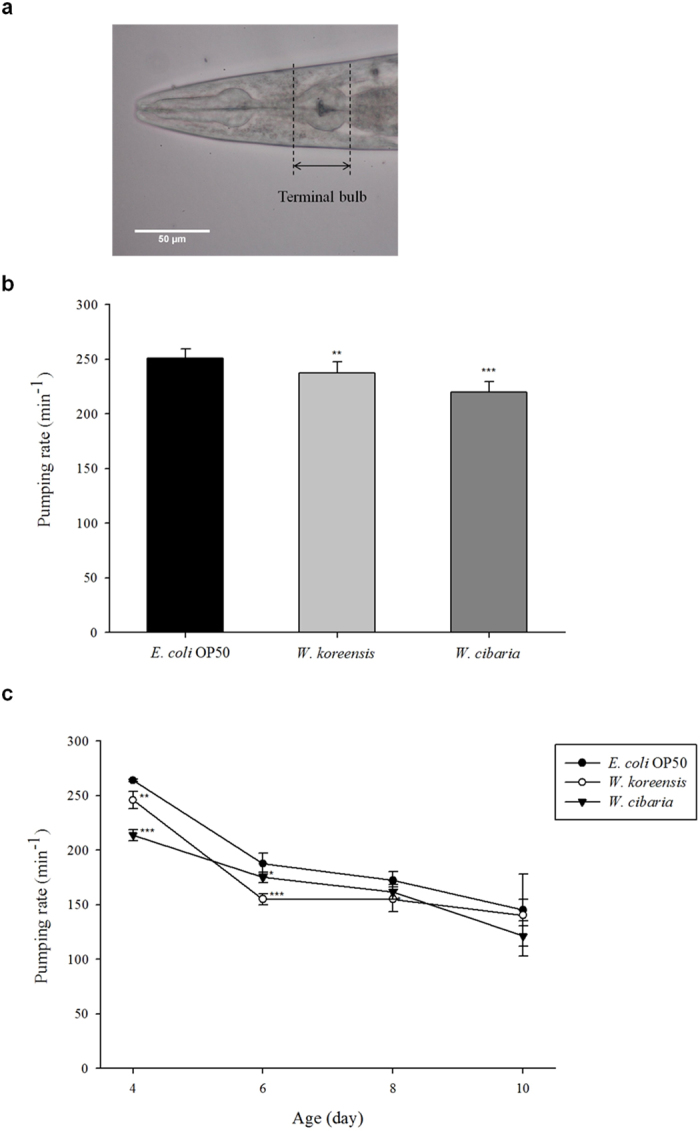
The pharyngeal pumping rate of *C. elegans* (N2) fed *E. coli* OP50 and *Weissella*. Pumping rate was measured in the terminal bulb (**a**). The scale bar represents 50 μm. (**b**) The pumping rate of 3-day-old worms, measured for 30 min after the worms were transferred to a fresh mNGM plate seeded with each bacterial species. (**c**) The pumping rate in ageing worms, determined from the mean of 20 worms for each bacterial species. Significant differences shown are relative to *E. coli* OP50 (^*^p < 0.05, ^**^p < 0.01, ^***^p < 0.001).

We next determined the level of food intake by measuring the pharyngeal pumping rate. Lowering the pharyngeal pumping rate can induce dietary restriction by limiting feeding (similar to what occurs in *eat-2* mutants)^18^. The pumping rate was measured 30 min after transferring worms to *E. coli* OP50 or *Weissella* plates by counting the number of contractions in the terminal bulb of the pharynx (Fig. 5a) for 1 min. The pumping rate of worms grown on the *Weissella* lawn decreased significantly (*p* < 0.05) after 3 days (Fig. 5b). The pumping rate was then measured every 24 h from day 4 to day 10, during which the pumping rate of the *Weissella* group was lower than that of the *E. coli* group (Fig. 5c). Regardless of the bacterial species, the pumping rate decreased with age.

Because ATP level is related to dietary restriction^19^ as well as the AMPK pathway, we measured ATP levels and found that worms fed *W. koreensis* and *W. cibaria* had a 23.4% and 87.0% decrease in ATP, respectively, compared with the worms fed *E. coli* OP50 (Fig. S2). Therefore, *Weissella* significantly affects ATP content in *C. elegans,* especially *W. cibaria*, which dramatically decreased ATP production.

### *C. elegans* food preference

*C. elegans* is known to exhibit preferences for specific bacterial strains^20^. A chemotaxis assay was performed to prove that the lifespan extension effect of *Weissella* was not derived from a preference between *E. coli* OP50 and *Weissella*. The results showed that *C. elegans* appeared to prefer *E. coli* OP50 to *Weissella* at 30 min after feeding the test strains; however, the numbers of worms in the circle (see Fig. S3) around *E. coli* OP 50 or *Weissella* were similar (more than 300 worms) in each test at 60 min after feeding the test strains, which means that *C. elegans* did not show any preference between *E. coli* OP50 and *Weissella* by 60 min (Table S2). Interestingly, many worms appeared to prefer lactic acid (125.0 ± 4.00) at 30 min after feeding on the test compounds, and again, the numbers of worms in the circle were similar (more than 300 worms) in each test at 60 min after feeding on lactic acid or 95% ethanol.

### Expression of lifespan extension-related genes

We investigated the expression of age-related genes, including those involved in diet restriction in *C. elegans*, to determine the mechanism of lifespan extension in *Weissella-*fed *C. elegans*. Quantitative real-time (qRT)-PCR was performed on the genes shown in Table S3. In *Weissella-*fed worms, *daf-16*, *sod-3*, *jkk-1*, *jnk-1* and *aak-2* were significantly overexpressed (Fig. S4), especially in worms fed *W. cibaria*, where all genes except *daf-2* were up-regulated more than two-fold, with *daf-16* and *sod-3* upregulated 3.9-fold and 2.3-fold, respectively, compared with the *E. coli* OP50 control group. Meanwhile, in the *W. koreensis* group, *daf-16*, *sod-3*, *jkk-1*, *jnk-1* and *aak-2* expression increased, but to a lesser extent than the *W. cibaria* group, and there were no significant increases in *daf-2* and *hif-1* expression. The other genes observed in this study did not show significant increases compared with the worms fed *E. coli* OP50 (Fig. S4).

### Effect of *Weissella* species on nuclear translocation of DAF-16

**Figure 6:**
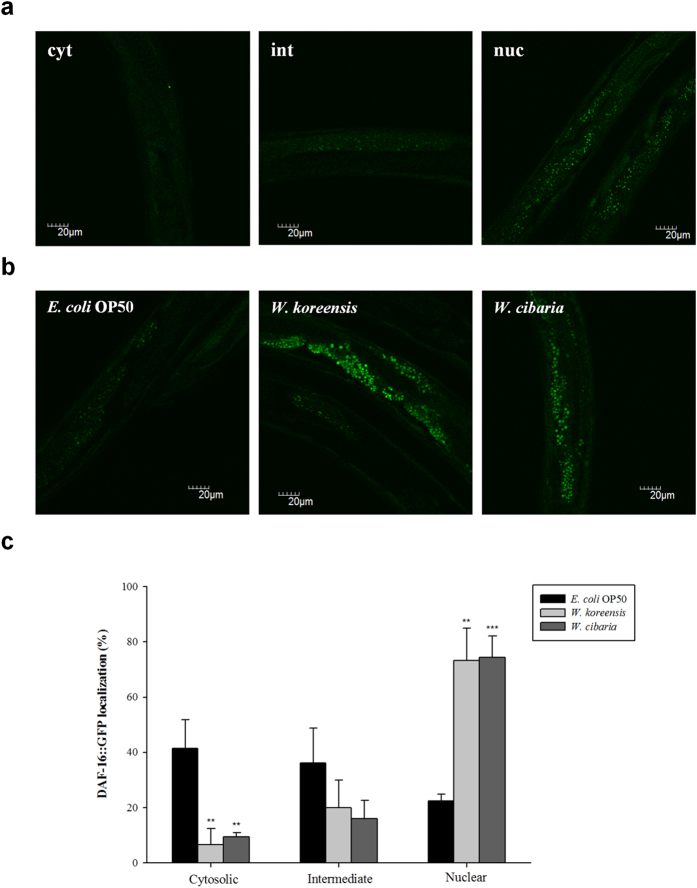
*Weissella* species induced nuclear translocation of DAF-16::GFP. (**a**) Depending on the degree of nuclear GFP fluorescence, three different states of translocation of DAF-16::GFP from the cytoplasm into cell nuclei can be distinguished: cytosolic (cyt, left; no nuclear GFP fluorescence), intermediate (int, centre; weak nuclear GFP fluorescence), and nuclear (nuc, right; strong nuclear GFP fluorescence) DAF-16::GFP localization. (**b**) Representative images of transgenic strain CF1407 fed with *E. coli* OP50 (left), *W. koreensis* (centre) and *W. cibaria* (right). Scale bar = 20 μm. (**c**) Nuclear localization of DAF-16 by *Weissella* species. Values shown are the mean ± SD from 90 worms for each bacterial species. Significant differences shown are relative to *E. coli* OP50 (^**^p < 0.01, ^***^p < 0.001).

We analysed the nuclear localization of DAF-16 by using transgenic strains expressing the fusion protein DAF-16::GFP (Fig. 6b). *Weissella* species induced an increase of nuclear DAF-16::GFP translocation (*E. coli* OP50: 22.4%; *W. koreensis*: 73.3%; *W. cibaria*: 75.0%) and a reduction of the cytosolic DAF-16::GFP fraction (*E. coli* OP50: 41.5%; *W. koreensis*: 6.7%; *W. cibaria*: 9.4%) (Fig. 6c).

### Lifespan extension in *C. elegans* mutants

To confirm the role of the aforementioned genes in lifespan extension, *C. elegans* loss-of-function mutants for each of these genes were fed *Weissella* or *E. coli* OP50, and their longevity was assessed. *Weissella*-fed mutant worms with loss-of-function mutations in *daf-16*, *aak-2* or *jnk-1* showed no extension in lifespan compared with the *E. coli* control group, which suggests an essential role for these genes in the increased longevity of *Weissella*-fed worms (Table 1, Fig. S5). Unexpectedly, the lifespans of *sod-3*, *jkk-1, skn-1, hif-1* and *daf-2* mutants still increased when fed *Weissella*. The finding that the loss of *daf-2* does not affect lifespan extension suggests that the IIS pathway is not involved in *Weissella-*dependent lifespan extension. Taken together with the qRT-PCR results, these above results suggest that *daf-16* may be the key in promoting longevity in *Weissella-*fed worms.

## Discussion

Although *W. koreensis* and *W. cibaria* isolated from kimchi might be candidate probiotics, little information, including the lifespan-extension effect of *Weissella* on *C. elegans*, is available on *Weissella* species in general. Previously, biomarkers of ageing such as lipofuscin, body size and locomotor activity have been studied to estimate pro-longevity in *C. elegans*. Feeding *Weissella* to *C. elegans* significantly increased MLS and was negatively associated with biomarkers of ageing. Feeding *Weissella* to *C. elegans* reduced lipofuscin accumulation, body size, brood size and pharyngeal pumping rate. Additionally, the locomotor activity of *C. elegans* fed with *Weissella* decreased more slowly than in worms fed with *E. coli* OP50. Body movement can be measured by the locomotory test, and the results of locomotory class are predictive of the remaining lifespan of *C. elegans* after 8 days of age^21^. Lipofuscin (referred to as age pigment) accumulates in post-mitotic cells and is therefore a marker of ageing in animals such as *C. elegans*^22^. Decreases in body size and pumping rate are related not only to ageing but also to dietary restriction, which extends lifespan by reducing brood size^23^. The inherent ability of nematodes to produce offspring, adult nematodes’ egg-laying activity and the ability of newly hatched nematodes to survive and develop can affect brood size. In this experiment, all *C. elegans* ate *E. coli* OP50 during the early larval stage, and therefore, the influence of *Weissella* on the inherent ability of nematodes to produce offspring could be excluded. During the experiment, there were no remarkable differences in the numbers of dead eggs and progeny that failed to develop between *E. coli* OP50- and *Weissella*-fed worms. Therefore, *Weissella* might mainly affect the egg-laying activity of adult nematodes.

Moreover, dietary restriction limits ATP production in *C. elegans*, and ATP levels significantly decreased in *Weissella-*fed *C. elegans* compared with *E. coli* OP50-fed worms. These results suggest that *Weissella* may extend the lifespan of *C. elegans* through dietary restriction. According to the attraction assay, *C. elegans* initially appears to be more attracted to *E. coli* OP50 than to *Weissella*; however, *C. elegans* showed no preference between *E. coli* OP50 and *Weissella* by approximately 60 min. These results might be related to the fact that *C. elegans* rapidly change their behaviours and innate chemosensory preferences^24^. This means that dietary restriction was not linked to a repulsion of *C. elegans* towards *Weissella*.

While the biological effects of ROS on senescence and anti-senescence remain controversial^25^,^26^, ROS (by-products of respiration) are generally believed to be harmful to biological processes. However, it has been reported that ROS production induces enzymes that detoxify oxygen radicals, to defend against ROS damage^27^. In this study, ROS production significantly decreased in worms fed *W. koreensis*, yet it increased in worms fed *W. cibaria* compared with controls. This may have occurred because *Weissella* species might produce different types of ROS that are more or less reactive to 2,7-dichlorofluorescein diacetate (DCF-DA). Other mechanisms could also have affected the ROS results; for example, several types of inhibitors might affect the ROS levels in worms fed *W. koreensis* or *W. cibaria*. Furthermore, differences in ROS levels between *W. koreensis* and *W. cibaria* might be derived from the limitations of methods using DCF-DA^28^.

In *C. elegans*, dietary restriction resulted in low-level ROS production and increased lifespan^29^. Although the factor(s) involved in elevating ROS levels in *W. cibaria-*fed *C. elegans* remain unknown, several positive effects have been reported regarding ROS and longevity in *C. elegans*. First, ROS induces the expression of superoxide dismutase (SOD), which detoxifies ROS^30^,^31^. Second, ROS promote the expression of *hif-1*, which also regulates lifespan. Although it is still not clear how HIF-1 is activated under low-oxygen conditions and which downstream genes facilitate lifespan extension, an increase in ROS levels extends the lifespan of *C. elegans* by stabilizing HIF-1^32^. Third, high levels of ROS induce phosphorylation and the subsequent activation of JNKs^33^, which may also occur in *C. elegans*. It still remains controversial whether dietary restriction extends lifespan by reducing ROS production or by increasing ROS defences and repair^34^. Likewise, ROS levels in worms fed with *W. cibaria* and *W. koreensis* increased and decreased, respectively, compared with the worms fed with *E. coli* OP50. ROS production in *C. elegans* fed with *W. cibaria* was not consistent with the results for lipofuscin, which is a complex mixture of oxidized and cross-linked macromolecules including proteins, lipids and carbohydrates. Lipofuscin levels in worms fed with both *Weissella* species were significantly lower than in worms fed with *E. coli* OP50. Metabolic and detoxification activities change with age in *C. elegans*^35^. In addition, ROS levels were measured using 4-day-worms that fed on *Weissella* species for 24 h, while lipofuscin levels were measured using 14-, 16- and 18-day worms, which may have caused the inconsistency between ROS level and lipofuscin accumulation in *W. cibaria*-fed *C. elegans*. We could therefore not conclude what the effects of dietary restriction on ROS levels were from our data.

The results obtained using *W. koreensis* or *W. cibaria* were slightly different. In this study, *W. koreensis* did not affect *hif-1* gene expression. However, *W. cibaria* induced overexpression of *hif-1* and still increased the lifespan of *hif-1* mutants. DAF-16/FOXO might be actively relocalized in *hif-1*-mutants; it has been shown that *hif-1* mutants live longer than wild-type worms because knocking down *hif-1* triggers nuclear relocalization of DAF-16/FOXO^36^. The expression of *sod-3* increased in both *Weissella* groups, but to a lesser extent in the *W. koreensis* group. The lifespan of *sod-3* mutants fed *W. koreensis* and *W. cibaria* was extended compared with that of *sod-3* mutants fed *E. coli.* The results suggest that SOD-3 is not an essential target of either *Weissella* species for extending the lifespan of *C. elegans*. However, the overexpression of *sod-3* in the *W. cibaria*-fed worms might be related to the increase of ROS production in this group.

Pro-longevity related to dietary restriction is associated with many pathways, such as the TOR pathway, the AMPK pathway, the IIS pathway and sirtuins^14^. Among these pathways, DAF-16, which functions as a transcription factor, plays a major role in lifespan extension^37^ by regulating longevity, fat metabolism, stress response, free-radical detoxification and pathogen resistance. Meanwhile, suppression of DAF-2, which is related to the IIS pathway, extends lifespan by negatively regulating DAF-16^38^. In this study, however, *daf-16* expression increased in *Weissella-*fed *C. elegans* despite increased expression of *daf-2.* Therefore, lifespan extension was apparently not related to the IIS pathway. The stress-related genes *sod-3* and *hif-1* can also affect *C. elegans* lifespan^30^,^31^. The genes *jkk-1* and *jnk-1* are members of the JNK pathway^39^, and *aak-2* activates the AMPK pathway^16^. We used *C. elegans* loss-of-function mutants to investigate the relevant contributions of longevity-associated pathways to *C. elegans* lifespan; *jnk-1* and *aak-2* mutants were used to investigate the role of the JNK and AMPK pathways, respectively. We found that several age-related genes increased in *Weissella-*fed worms, and both *W. koreensis* and *W. cibaria* failed to extend lifespan in *C. elegans daf-16*, *aak-2* or *jnk-1* mutants, which suggests that these genes are essential to lifespan extension in *Weissella-*fed *C. elegans*. JNK-1 and AAK-2 modulate DAF-16 activity by phosphorylation; specifically, the JNK family, a subgroup of the MAPK superfamily, is a part of the signal transduction cascade activated by exposure to environmental stress and cytokines^40^. In *C. elegans*, JNK-1 directly interacts with DAF-16 to mediate nuclear translocation of DAF-16, which triggers upregulation of several stress- and damage-response genes^39^. Similarly, AAK-2, which is one of the two α-catalytic subunits of AMPK, has recently been found to increase worm longevity^41^. AMPK is an important mediator of dietary restriction on longevity that acts via DAF-16/FOXO^16^ and is independent of the IIS pathway because DAF-16 is directly phosphorylated by AMPK^16^. The *aak-2* gene encodes low-energy-sensing AMPK, and therefore, it is linked to dietary restriction. AMPK/*aak-2* is necessary to mediate lifespan extension via dietary restriction^42^. The lifespan of the *C. elegans aak-2* mutant was not extended, and the expression of the *aak-2* gene significantly increased with feeding on *Weissella*. In *Weissella-*fed *C. elegans*, ATP levels significantly decreased, which is consistent with the activation of the AMPK pathway. Our results demonstrate that *Weissella* species promote longevity in *C. elegans* by increasing the expression of *daf-16* via the JNK and AMPK pathways.

**Figure 7:**
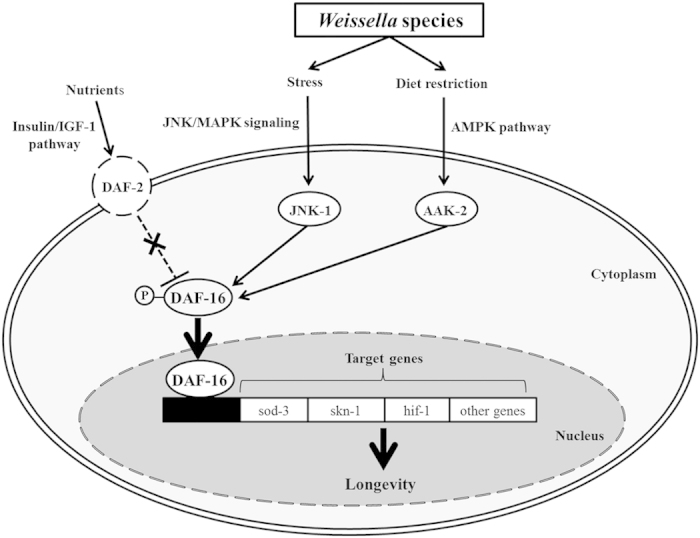
Mechanism predicted to be involved in the lifespan extension effect of *Weissella*. JNK-1 and AAK-2 activate DAF-16 through the JNK and AMPK pathways, respectively. Activated DAF-16 affected *sod-3*, *hif-1* and other genes related to longevity. The insulin/IGF-1 receptor, DAF-2, was not related to the lifespan extension of *C. elegans* by *Weissella*.

The mechanism of lifespan extension of *Weissella*-fed *C. elegans* was different from those of worms fed *B. infantis*^9^ and *L. rhamnosus*^10^. Based on qRT-PCR and mutant survival data, we predicted the pathways involved in lifespan extension of *Weissella*-fed *C. elegans* (Fig. 7). Briefly, *Weissella* promoted *C. elegans* longevity by inducing dietary restriction and stress response and, consequently, downstream expression of *daf-16* via the AMPK and JNK pathways. We do not know if *Weissella* is lower in calories or less nutritious compared with *E. coli* OP50. Interestingly, although brood size decreased, the brooding period increased in *Weissella*-fed *C. elegans*. In this study, *C. elegans* was allowed to feed freely on the food source (*E. coli* OP50 or *Weissella*); thus, dietary restriction was not artificially induced. Dietary restriction in *C. elegans* was not derived from a repulsion to *Weissella* and was not associated with lactic acid produced by *Weissella*.

Metchnikoff hypothesized that lactic acid bacteria are important for human health and longevity on the basis of the longevity of Bulgarians who eat plenty of yogurt^43^. It is possible that similar mechanisms of pro-longevity exist in *C. elegans*. Additionally, dietary restriction is known to extend lifespan and to retard age-related health declines in a number of different species, including rodents, worms, yeast and possibly primates^44^. It is unclear whether dietary restriction affects other animals in the same way, but it is possible that the mechanisms identified in this study may apply to other species including humans. Finally, the JNK and AMPK pathways stimulate autophagy under dietary restriction, and therefore, further studies on the possible relation between autophagy in lifespan extension of *C. elegans* by *Weissella* are required. In addition, in the present study, a subset of genes known to extend lifespan were investigated; thus, further studies are needed to investigate the whole genome of *C. elegans* to reveal other possible factors and/or pathways contributing to the lifespan extension of *C. elegans* by *Weissella*.

## Methods

### Bacterial strains and culture conditions

*W. koreensis* KACC 11853 and *W. cibaria* KACC 11845 were obtained from the Korean Agricultural Culture Collection (KACC) and used as test food sources for nematodes. *E. coli* OP50 was provided by the Caenorhabditis Genetics Center, University of Minnesota (CGC) and used as a control food source. *E. coli* OP50 was grown in Luria-Bertani (LB) broth (Difco, Detroit, MI, USA) at 37 °C for 18**−**24 h with shaking. *Weissella* strains were grown at 30 °C for *W. koreensis* and 37 °C for *W. cibaria* in de Man, Rogosa and Sharpe (MRS) broth (Difco) for 24 h without shaking. Bacteria were harvested by centrifugation at 3,000 × *g* for 10 min and washed in sterile M9 buffer. Then, bacteria were adjusted to a final concentration of 0.1 mg (wet weight) per microlitre in M9 buffer^8^.

### Nematodes and growth conditions

*C. elegans* Bristol strain N2 (wild-type) and mutant strains were provided by the CGC. Bristol strain N2 was used for all measurements except the DAF-16 localization assay and the longevity assay with the mutant strains. The mutants used for lifespan measurements were CF1038 daf-16 (mu86), RB754 aak-2 (ok524), EU1 skn-1 (zu67), GA186 sod-3 (tm760), VC8 jnk-1 (gk7), ZG596 hif-1 (ia7), CB1370 daf-2 (e1370) and KU2 jkk-1 (km2). CF1407 daf-16 (mu86) I; muIs 71 [pKL99(daf-16Ap::GFP::daf-16A(bKO)) + pRF4(rol-6)] was used for the DAF-16 localization assay. Nematodes were maintained and propagated at 25 °C according to standard techniques^45^. The bacterial suspension was spread on peptone-free modified nematode growth medium (mNGM) in 90-mm diameter plates to feed worms. To exclude the possibility of nematocidal effects of nutrients in the bacterial growth medium, every experiment was performed on mNGM plates^9^. Eggs were obtained from adult worms after being exposed to a sodium hypochlorite-sodium hydroxide solution as previously described^46^. The egg suspension in M9 buffer was incubated overnight at 25 °C to allow eggs to hatch, and the suspension of L1 stage worms was centrifuged at 1,200 × *g* for 2 min. After removing the supernatant, the remaining larvae were transferred onto fresh mNGM plates seeded with *E. coli* OP50 and incubated at 25 °C for two days to synchronize pubescence. All experiments were conducted with 3-day-old young-adult (day 1 of adulthood) wild-type worms (except for mutant survival tests).

### Longevity assay

For the longevity assay, mNGM/FUdR plates were produced by supplementing with 5-fluoro-2′-deoxyuridine (FUdR, Sigma Aldrich) (50 μM)^47^. *E. coli* OP50, *W. koreensis* and *W. cibaria* cells in M9 buffer (20 mg wet weight) were spread on mNGM/FUdR plates (90-mm diameter). The longevity assay was started at the L4 stage of N2 nematodes and mutants, after which the worms were transferred to plates with a platinum wire. For each assay, 30 worms were assayed on three plates (10 worms per plate) for each bacterial species. The plates were incubated at 25 °C, and live and dead worms were counted every 24 h. A worm was considered dead when it failed to respond to a gentle touch with a platinum wire picker. Worms showing abnormal death, such as hatched progeny inside the body, vulva explosion, or death as a result of adhering to the wall of the plate were excluded from the lifespan analysis. During the assay, worms being tested were transferred to fresh mNGM plates every day for the first 3 days and once a week for the rest of experiment to maintain a sufficient food source. The longevity assay was performed at least three times independently. The mean lifespan was estimated using Eq. (1)^48^:


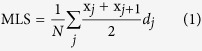


where *j* is the age (day), *d*_*j*_ is the number of worms that died in the age interval (x _j_, x _j+1_), and *N* is the total number of worms. The standard error (SE) of the estimated mean lifespan (MLS) was calculated using Eq. (2):


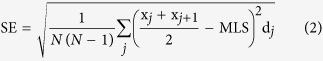


### Quantification of ROS in *C. elegans*

Total ROS levels were quantified in whole worms using 2,7-dichlorodihydro-fluorescein-diacetate (H_2_-DCF-DA) (Sigma-Aldrich). ROS levels were determined according to previously performed protocols with slight modifications^49^. Briefly, worms were fed on each bacterial lawn for 24 h and collected with M9 buffer. Bacteria were removed by washing three times with M9 buffer, and worms were resuspended in M9 buffer. To avoid overestimation of ROS levels, whole worms were used^50^. A 50-μl aliquot of the suspension was transferred into each well of a black 96-well plate, and a 50-μl aliquot of the freshly prepared 100 μM H_2_-DCF-DA solution was added, resulting in a final concentration of 50 μM. Four wells were used per sample. There were control wells containing nematodes from each bacterial lawn without H_2_-DCF-DA and containing H_2_-DCF-DA without nematodes. After the addition of H_2_-DCF-DA, the basal fluorescent signal from each well was immediately measured with a fluorescence microplate reader (SpectraMAX GEMINI EM, Molecular Devices) at excitation and emission wavelengths of 485 nm and 520 nm, respectively. After the initial reading, the plate was incubated at 25 °C for 1 h, and the fluorescence intensity was measured at the same wavelength. The change in fluorescence was determined by subtracting the initial value from the final value for each well including control wells. One millilitre of the initial worm suspension from each sample was used for protein quantification to normalize the fluorescence signal. Three independent experiments were performed.

### Lipofuscin accumulation assay

The autofluorescence of intestinal lipofuscin was measured as an index of senescence between days 10 and 18 of adulthood. Randomly selected worms from the plate lawned with *E. coli* OP50 or *Weissella* species were washed three times with M9 buffer. Worms were then placed onto a 5% agar pad coated with 10 mM sodium azide (Junsei Chemical, Tokyo, Japan) in M9 buffer to induce anaesthesia. Lipofuscin autofluorescence images were taken using blue excitation light (405**−**488 nm), a DAPI (4′,6-diamidino-2-phenylindole) channel of laser confocal scanning microscope (Olympus Ix81-FV1000, Japan)^51^. Fluorescence was quantified on days 14, 16 and 18 using ImageJ software (National Institutes of Health, Bethesda, MD, USA) to determine the lipofuscin levels. Three independent experiments were performed with over 30 worms for each bacterial species on each day.

### Locomotory scoring

The locomotor activity of worms at different ages was examined using a scoring method described in previous reports^21^,^52^. Worms were classified as class “A” when they showed spontaneous movement or vigorous locomotion in response to prodding; class “B” worms did not move unless prodded or appeared to have uncoordinated movement; class “C” worms moved only their head and/or tail in response to prodding; class “D” worms were dead. Experiments were repeated three times independently, and at least 100 worms were scored for each bacterial species.

### Measurement of body size

Three-day-old adult worms (L4 stage) were transferred to mNGM plates (60 mm diameter) covered with 5 mg (wet weight) *Weissella* or *E. coli* OP50 cells in M9 buffer. The plates were incubated at 25 °C, and the body size of live worms was measured every 24 h until 7 days of age. Five worms per bacterial species were assayed using five plates (one worm per plate). Images of worms were taken with a stereomicroscope (Olympus SZ61) and a ToupCam (UCMOS05100KPA). Images were analysed using ImageJ software. In this system, the area of a worm’s projection was estimated automatically and used as indices of body size^7^. Three independent experiments were performed with 20 worms for each bacterial species.

### Brood size

L4-stage worms were transferred to mNGM plates coated with *Weissella* or *E. coli* OP50 and incubated at 25 °C. The parental animals were transferred daily to fresh mNGM plates (60 mm diameter) until the end of the reproductive period. The progeny of each animal were counted at the L2 or L3 stage. Ten worms per bacterial species were assayed using five plates (two worms per plate), and the test was performed three times^9^.

### Pharyngeal pumping rate

Pumping assays were performed on mNGM plates with bacterial lawns at room temperature. After 30 min, L4-stage worms (3 days old) were transferred to bacteria-seeded mNGM plates, and the number of contractions in the terminal bulb of the pharynx was counted for 1 min using an Olympus CKX41 inverted microscope (×400). The worms were incubated at 25 °C, and the worms’ pumping rate on each bacterial lawn was measured every 24 h^8^. Three independent experiments were performed with 20 worms for each bacterial species on each day.

### Quantification of ATP in *C. elegans*

Four-day-old adult worms fed on each bacterial lawn for 24 h were collected and washed three times in M9 buffer. Worm pellets were treated with three freeze/thaw cycles and boiled for 15 min to release ATP and destroy ATPase activity. After that, worms were centrifuged at 12,000 × *g* for 10 min at 4 °C, and ATP levels were quantified with the ATPlite kit according to the manufacturer’s instructions (PerkinElmer, USA). Luminescence was measured with a fluorescence microplate reader (Molecular Devices, SpectraMAX GEMINI EM). The soluble protein concentration of the same preparation was measured using the Bradford assay, and the ATP content value was normalized against the protein level^53^. Three independent experiments were performed.

### Attraction assay

To examine the chemotactic activity of worms towards *Weissella*, 95% ethanol (Samchun chemical, Korea), lactic acid (Samchun chemical) and M9 buffer, we designed a chemotaxis assay with slight modification of the methods of Bargmann^54^ and Beale^55^. Bacterial food or other material was spotted onto the centre of the 90-mm diameter mNGM plates (Fig. S3), and over 1,000 worms were placed 10 mm from the sides of the plates. After 30 min and 60 min of incubation at 25 °C, the number of worms in the 30 mm-diameter-circle in the centre of each plate, including each bacterial lawn, was counted. Three independent experiments were performed.

### RNA isolation and quantitative real-time polymerase chain reaction (RT-PCR)

Worms fed on *E. coli* OP50 or *Weissella* for 24 h were collected in M9 buffer, and total RNA was isolated from whole worms as previously reported^56^. Total RNA was converted to cDNA using the RevertAid First Strand cDNA Synthesis kit according to the manufacturer’s instructions (Thermo Scientific), followed by quantitative RT-PCR (qRT-PCR) using the SYBR green (KAPA Biosystems, USA) and StepOnePlus real-time PCR system (Applied Biosystems). Primers were designed using Primer 3 software^57^. The primer sequences are shown in Table S3. Reactions were initiated at 95 °C, followed by 40 cycles of 95 °C for 20 s, 56 °C for 20 s and 72 °C for 30 s, followed by melt curve analysis. Relative expression levels were calculated using the 2^−ΔΔCT^ method^58^. The control gene *act-2* was used to normalize gene expression data^59^. Three independent experiments were performed.

### DAF-16 localization assay

The transgenic strain CF1407 *daf-16* (mu86) I; *muIs* 71 [pKL99(daf-16Ap::GFP::daf-16A(bKO)) + pRF4(rol-6)] was used to detect the intracellular localization of GFP-tagged DAF-16 protein. Randomly selected 4-day-old worms that were fed on plates lawned with *E. coli* OP50 or *Weissella* species for 24 h were washed three times with M9 buffer. Worms were then placed onto 5% agar pads coated with 10 mM sodium azide (Junsei Chemical, Tokyo, Japan) in M9 buffer to induce anaesthesia. GFP images were taken using green excitation light (460**−**495 nm), a GFP (green fluorescence protein) channel of a laser confocal scanning microscope (Olympus Ix81-FV1000, Japan). Localization of DAF-16 GFP from the cytoplasm into the cell nuclei was examined by analysing the degree of nuclear GFP fluorescence. Three different states can easily be distinguished (no, weak, or strong nuclear GFP fluorescence), which are related to a cytoplasmic, intermediate, or nuclear location of DAF-16-GFP^60^. The degree of nuclear translocation of DAF-16 was evaluated by counting the number of worms showing either weak or strong nuclear GFP fluorescence. Three independent experiments were performed with over 90 worms for each bacterial species.

### Statistical analysis

The nematode survival rate was calculated by the Kaplan−Meier method, and the significance of survival differences was tested using the Log-rank test^8^. Differences in lipofuscin levels were assessed using the Mann−Whitney U test^9^. One-way analysis of variance (ANOVA) with the post hoc Tukey test was used to compare the effect of *Weissella* on pharyngeal pumping rates, and ANOVA with Duncan’s test was used to compare chemotaxis towards different food sources. Results were considered significant if *p* < 0.05. In other experiments, the means of the *E. coli* OP50 and *Weissella* group values were determined using Student’s t-test. *P*-values ≤ 0.05 were considered statistically significant, and error bars depict the standard deviation.

## Additional Information

**How to cite this article**: Lee, J. *et al.* Elucidating the Mechanism of *Weissella*-dependent Lifespan Extension in *Caenorhabditis elegans*. *Sci. Rep.* **5**, 17128; doi: 10.1038/srep17128 (2015).

## Supplementary Material

Supplementary Information

## Figures and Tables

**Table 1:** The mean lifespan of wild-type *C. elegans* (N2) and *C. elegans* loss-of-function mutants when fed *E. coli* OP50, *W. koreensis* and *W. cibaria*.

Nematode type	Food source (bacteria)	No. total worms (dead/censored)	MLS ± SE (days)
N2 (wild-type)	*E. coli* OP50	136 (120/16)	15.4 ± 0.21
	*W. koreensis*	150 (105/45)	17.1 ± 0.25 ^***^
	*W. cibaria*	169 (125/44)	16.7 ± 0.32 ^***^
CF1038 *daf-16 (mu86)*	*E. coli* OP50	169 (158/11)	13.3 ± 0.13
	*W. koreensis*	174 (143/31)	12.9 ± 0.17
	*W. cibaria*	182 (159/23)	13.4 ± 0.15
RB754 *aak-2 (ok524)*	*E. coli* OP50	168 (154/14)	12.2 ± 0.16
	*W. koreensis*	180 (154/26)	11.8 ± 0.15
	*W. cibaria*	165 (148/17)	12.0 ± 0.16
VC8 *jnk-1 (gk7)*	*E. coli* OP50	167 (158/9)	15.5 ± 0.21
	*W. koreensis*	169 (156/13)	15.1 ± 0.23
	*W. cibaria*	170 (155/15)	15.5 ± 0.21
KU2 *jkk-1 (km2)*	*E. coli* OP50	152 (149/3)	15.2 ± 0.25
	*W. koreensis*	160 (135/25)	17.5 ± 0.31 ^***^
	*W. cibaria*	161 (142/19)	18.5 ± 0.31 ^***^
CB1370 *daf-2 (e1370)*	*E. coli* OP50	189 (163/26)	25.5 ± 0.58
	*W. koreensis*	204 (166/38)	33.0 ± 0.75 ^***^
	*W. cibaria*	205 (170/35)	31.1 ± 0.78 ^***^
GA186 *sod-3 (tm760)*	*E. coli* OP50	169 (158/11)	14.8 ± 0.19
	*W. koreensis*	188 (154/34)	15.1 ± 0.23 ^*^
	*W. cibaria*	184 (161/23)	16.2 ± 0.21 ^***^
ZG596 *hif-1 (ia7)*	*E. coli* OP50	167 (154/13)	17.0 ± 0.22
	*W. koreensis*	148 (128/20)	18.6 ± 0.32 ^***^
	*W. cibaria*	175 (156/19)	17.9 ± 0.25 ^**^
EU1 *skn-1 (zu67)*	*E. coli* OP50	139 (129/10)	14.4 ± 0.24
	*W. koreensis*	142 (121/21)	15.7 ± 0.23 ^*^
	*W. cibaria*	142 (131/11)	17.6 ± 0.21 ^***^

*P*-values were calculated using the log-rank test. *P*-values were calculated relative to controls (*E. coli* OP50); ^*^p < 0.05, ^**^p < 0.01, ^***^p < 0.001.
