# Supplementary material for: Elucidating the Mechanism of Weissella-dependent Lifespan Extension in Caenorhabditis elegans
Source: Sci Rep. 2015 Nov 25;5:17128. doi: 10.1038/srep17128 (PMC4658530; doi:10.1038/srep17128)
Supplement: Supplementary Information [file srep17128-s1.pdf]

Supplementary Information

Elucidating the Mechanism of *Weissella*-dependent Lifespan Extension in *Caenorhabditis elegans*

Jiyun Lee, Gayeung Kwon, Young-Hee Lim

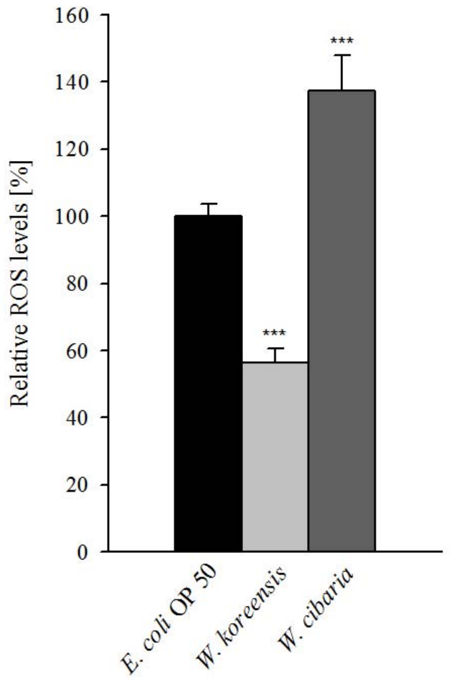

Fig. S1. The effect of *Weissella* on reactive oxygen species (ROS) levels in *C. elegans* (N2). The relative formation of ROS at 24 h after being fed on *Weissella* was measured. Differences shown are relative to *E. coli* OP50 ($^{***}p < 0.001$).

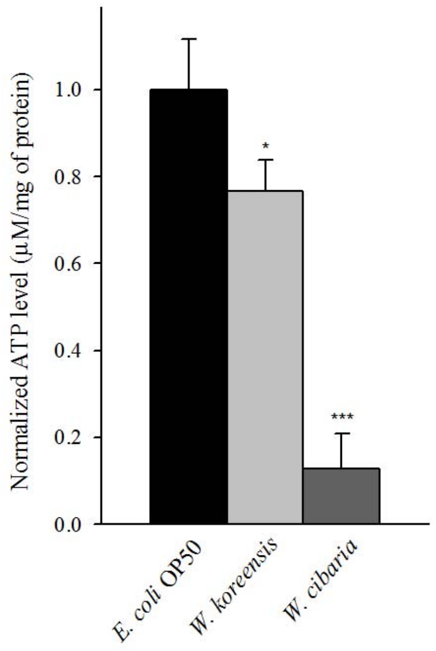

Fig. S2. ATP levels in *C. elegans* (N2) fed *E. coli* OP50 or *Weissella*. Values shown are the mean ± S.D. Significant differences shown are relative to *E. coli* OP50 ($^{*}p < 0.05$, $^{***}p < 0.001$).

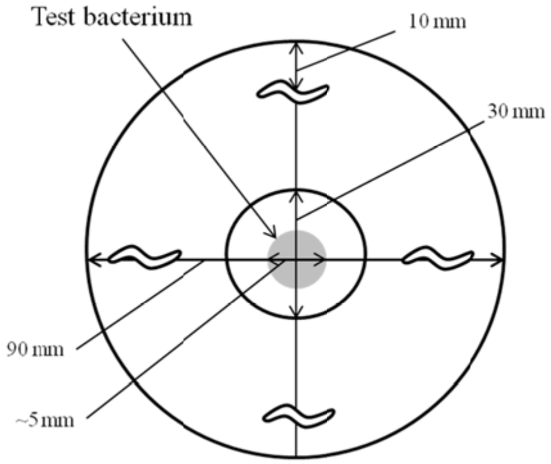

Fig. S3. Schematic representation of plates used for the modified chemotaxis-assays. Bacterial food or other material was spotted on the centre of the 90-mm diameter mNGM plates and over 1000 worms were placed 10 mm from the side of the plates.

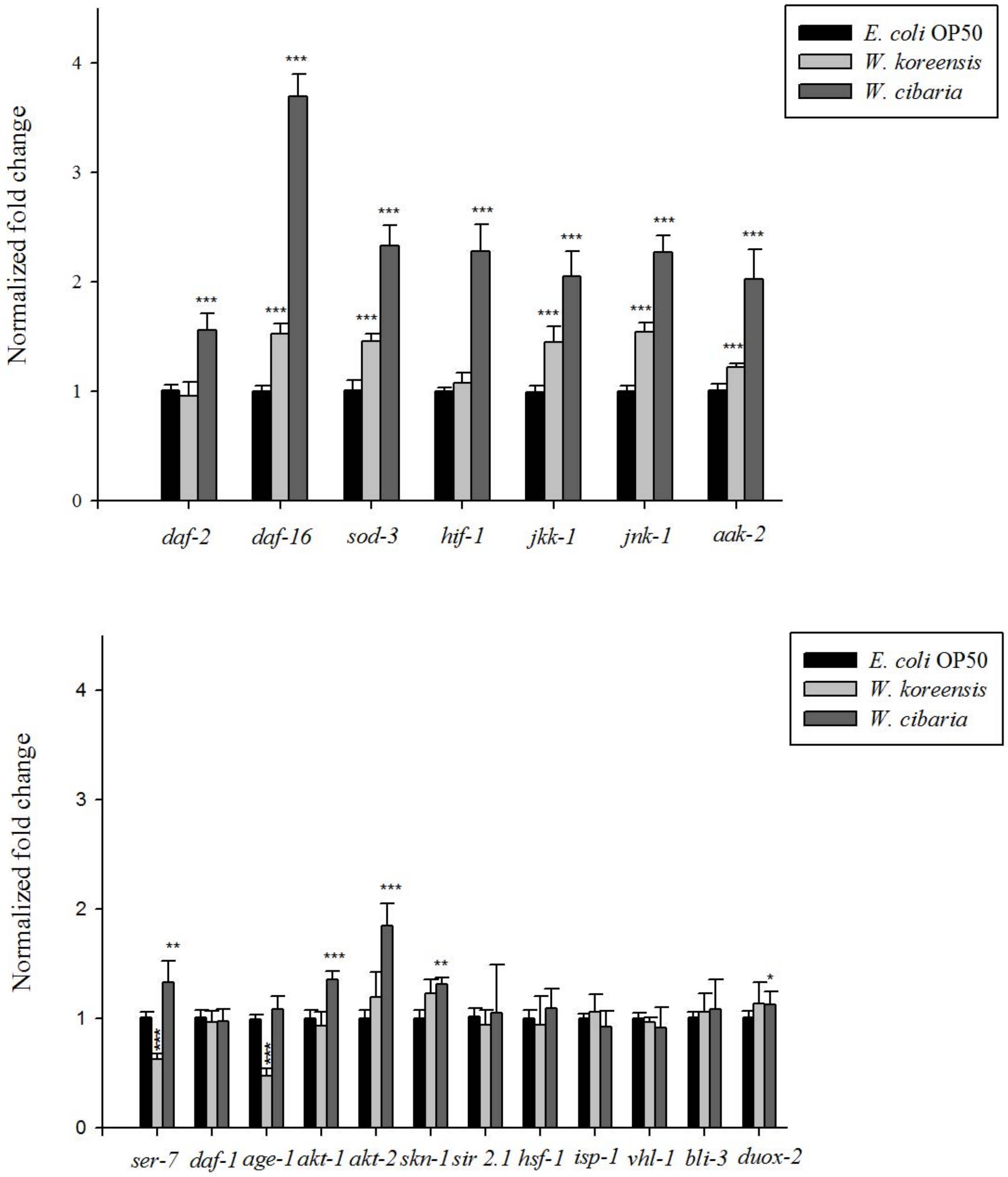

Fig. S4. The expression of age-related genes in ageing worms (N2) fed *E. coli* OP50 or *Weissella*. Significant differences shown are relative to *E. coli* OP50 ($^{*}p < 0.05$, $^{**}p < 0.01$, $^{***}p < 0.001$).

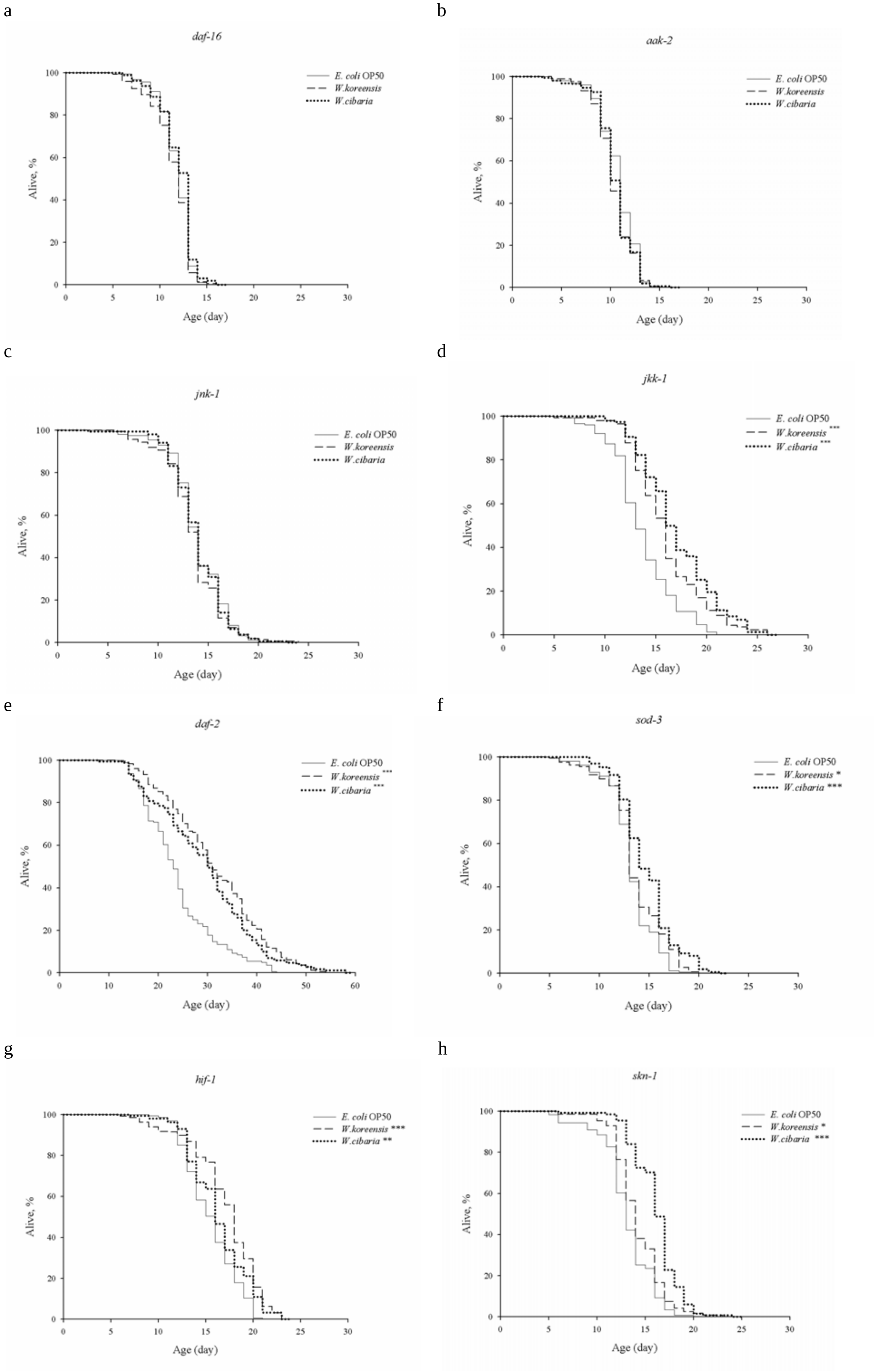
a
daf-16
E. coli OP50
W.koreensis
W.cibaria
Alive, %
Age (day)
b
aak-2
E. coli OP50
W.koreensis
W.cibaria
Alive, %
Age (day)
c
jnk-1
E. coli OP50
W.koreensis
W.cibaria
Alive, %
Age (day)
d
jkk-1
E. coli OP50
W.koreensis ***
W.cibaria ***
Alive, %
Age (day)
e
daf-2
E. coli OP50
W.koreensis ***
W.cibaria ***
Alive, %
Age (day)
f
sod-3
E. coli OP50
W.koreensis *
W.cibaria ***
Alive, %
Age (day)
g
hif-1
E. coli OP50
W.koreensis ***
W.cibaria **
Alive, %
Age (day)
h
skn-1
E. coli OP50
W.koreensis *
W.cibaria ***
Alive, %
Age (day)

Fig. S5. Influence of *Weissella* on the lifespans of *C. elegans* mutants. Survival curves of (a) *daf-16 (mu86),* (b) *aak-2 (ok524),* (c) *jnk-1 (gk7),* (d) *jkk-1(km2),* (e) *daf-2 (e1370),* (f) *sod-3 (tm760),* (g) *hif-1 (ia7)* and (h) *skn-1 (zu67)* mutant worms fed *E. coli* OP50 and *Weissella* species; $^{*}p < 0.05$, $^{**}p < 0.01$, $^{***}p < 0.001$.

Table S1. The mean lifespans of wild-type *C. elegans* (N2) and *C. elegans* mutants. All three independent lifespan data and *p* values are shown.

Nematode type	Food source (bacteria)	Trial	No. total worms (dead/censored)	MLS ± SE (days)	*P*-value
N2	*E. coli* OP50	1	31 (29/2)	15.3 ± 0.46	
		2	45 (45/0)	15.3 ± 0.33	
		3	60 (46/14)	15.6 ± 0.36	
		Total	136 (120/16)	15.4 ± 0.21	
	W. koreensis	1	46 (25/24)	17.5 ± 0.51	0.0005 ***
		2	46 (36/10)	16.2 ± 0.46	0.0301 *
		3	55 (44/11)	17.7 ± 0.35	0.0001 ***
		Total	150 (105/45)	17.1 ± 0.25	0.0000 ***
	W. cibaria	1	46 (31/15)	16.4 ± 0.76	0.0009 ***
		2	51 (35/16)	15.8 ± 0.26	0.0020 **
		3	72 (59/13)	17.1 ± 0.30	0.0022 **
		Total	169 (125/44)	16.7 ± 0.32	0.0000 ***
CF1038 *daf-16 (mu86)*	*E. coli* OP50	1	45 (41/4)	13.4 ± 0.31	
		2	63 (61/2)	13.3 ± 0.18	
		3	61 (56/5)	13.2 ± 0.41	
		Total	169 (158/11)	13.3 ± 0.13	
	W. koreensis	1	53 (50/3)	13.5 ± 0.30	0.5450
		2	60 (57/3)	12.8 ± 0.22	0.0628
		3	61 (36/25)	12.2 ± 0.84	0.1309
		Total	174 (143/31)	12.9 ± 0.17	0.3921
	W. cibaria	1	57 (48/9)	13.4 ± 0.32	0.7583
		2	61 (58/3)	13.6 ± 0.22	0.0696
		3	64 (53/11)	13.3 ± 0.67	0.5817
		Total	182 (159/23)	13.4 ± 0.15	0.0809
RB754 *aak-2 (ok524)*	*E. coli* OP50	1	45 (43/2)	12.1 ± 0.31	

		2	62 (58/4)	12.4 ± 0.20	
		3	61 (53/8)	12.2 ± 0.31	
		Total	168 (154/14)	12.2 ± 0.16	
	W. koreensis	1	53 (48/5)	11.4 ± 0.24	0.0463 *
		2	64 (50/14)	12.0 ± 0.19	0.1801
		3	63 (56/7)	12.0 ± 0.33	0.7935
		Total	180 (154/26)	11.8 ± 0.15	0.1161
	W. cibaria	1	55 (53/2)	12.6 ± 0.21	0.7595
		2	58 (51/7)	11.6 ± 0.29	0.0625
		3	52 (44/8)	11.7 ± 0.31	0.2324
		Total	165 (148/17)	12.0 ± 0.16	0.1974
VC8 *jnk-1 (gk7)*	*E. coli* OP50	1	46 (43/3)	15.2 ± 0.32	
		2	61 (59/2)	15.7 ± 0.35	
		3	60 (56/4)	15.5 ± 0.40	
		Total	167 (158/9)	15.5 ± 0.21	
	W. koreensis	1	49 (43/6))	14.0 ± 0.38	0.0431 *
		2	60 (54/6)	15.5 ± 0.40	0.9719
		3	60 (59/1)	15.4 ± 0.36	0.9729
		Total	169 (156/13)	15.1 ± 0.23	0.3392
	W. cibaria	1	50 (47/3)	15.7 ± 0.28	0.2510
		2	60 (49/11)	16.0 ± 0.44	0.5019
		3	60 (59/1)	14.8 ± 0.32	0.0539
		Total	170 (155/15)	15.5 ± 0.21	0.8097
KU2 *jkk-1 (km2)*	*E. coli* OP50	1	61 (60/1)	17.2 ± 0.37	
		2	41 (39/2)	13.6 ± 0.36	
		3	50 (50/0)	14.0 ± 0.36	
		Total	152 (149/3)	15.2 ± 0.25	
	W. koreensis	1	65 (51/14)	19.4 ± 0.60	0.0001 ***
		2	45 (36/9)	16.6 ± 0.46	0.0000 ***

		3	50 (48/2)	16.3 ± 0.32	0.0000 ***
		Total	160 (135/25)	17.5 ± 0.31	0.0000 ***
	W. cibaria	1	66 (53/13)	20.7 ± 0.53	0.0000 ***
		2	45 (39/6)	17.4 ± 0.51	0.0000 ***
		3	50 (50/0)	17.1 ± 0.35	0.0000 ***
		Total	161 (142/19)	18.5 ± 0.31	0.0000 ***
CB1370 *daf-2 (e1370)*	*E. coli* OP50	1	62 (50/12)	26.6 ± 0.97	
		2	66 (57/9)	24.6 ± 0.93	
		3	61 (56/5)	25.6 ± 1.11	
		Total	189 (163/26)	25.5 ± 0.58	
	W. koreensis	1	67 (52/15)	33.9 ± 1.31	0.0000 ***
		2	69 (59/10)	30.7 ± 1.03	0.0000 ***
		3	68 (55/13)	34.5 ± 1.51	0.0000 ***
		Total	204 (166/38)	33.0 ± 0.75	0.0000 ***
	W. cibaria	1	66 (60/6)	33.7 ± 1.31	0.0000 ***
		2	68 (50/18)	29.6 ± 1.30	0.0041 ***
		3	71 (60/11)	29.8 ± 1.37	0.0138 *
		Total	205 (170/35)	31.1 ± 0.78	0.0000 ***
GA186 *sod-3 (tm760)*	*E. coli* OP50	1	44 (40/4)	14.8 ± 0.43	
		2	60 (59/1)	14.7 ± 0.21	
		3	65 (59/6)	14.8 ± 0.36	
		Total	169 (158/11)	14.8 ± 0.19	
	W. koreensis	1	58 (53/5)	14.9 ± 0.31	0.6846
		2	60 (45/15)	13.8 ± 0.33	0.0599
		3	70 (56/14)	16.5 ± 0.42	0.0000
		Total	188 (154/34)	15.1 ± 0.22	0.0207 *
	W. cibaria	1	56 (53/3)	17.7 ± 0.40	0.0000 ***
		2	62 (54/8)	15.5 ± 0.21	0.0180 *
		3	66 (54/12)	15.4 ± 0.38	0.1829

		Total	184 (161/23)	16.2 ± 0.21	0.0000 ***
ZG596 *hif-1 (ia7)*	*E. coli* OP50	1	43 (41/2)	16.2 ± 0.35	
		2	64 (59/5)	16.4 ± 0.32	
		3	60 (54/6)	18.2 ± 0.40	
		Total	167 (154/13)	17.0 ± 0.22	
	W. koreensis	1	58 (43/15)	17.9 ± 0.72	0.0001 ***
		2	48 (45/3)	19.1 ± 0.43	0.0000 ***
		3	42 (40/2)	18.9 ± 0.49	0.2025
		Total	148 (128/20)	18.6 ± 0.32	0.0000 ***
	W. cibaria	1	54 (45/9)	16.9 ± 0.40	0.1649
		2	61 (54/7)	17.5 ± 0.37	0.0352 *
		3	60 (57/3)	19.0 ± 0.47	0.0295 *
		Total	175 (156/19)	17.9 ± 0.25	0.0015 **
EU1 *skn-1 (zu67)*	*E. coli* OP50	1	19 (18/1)	15.7 ± 0.35	
		2	60 (58/2)	14.8 ± 0.38	
		3	60 (53/7)	13.6 ± 038	
		Total	139 (129/10)	14.4 ± 0.24	
	W. koreensis	1	21(21/0)	16.0 ± 0.48	0.3402
		2	61 (53/8)	15.9 ± 0.34	0.0786
		3	60 (47/13)	15.4 ± 0.42	0.0926
		Total	142 (121/21)	15.7 ± 0.23	0.0152 *
	W. cibaria	1	20 (17/3)	18.4 ± 0.43	0.0002 ***
		2	60 (56/4)	18.1 ± 0.34	0.0000 ***
		3	62 (58/4)	17.0 ± 0.36	0.0000 ***
		Total	142 (131/11)	17.6 ± 0.21	0.0000 ***

p value versus control (*E. coli* OP50); $^{*}p < 0.05$, $^{**}p < 0.01$, $^{***}p < 0.001$

Table S2. Modified chemotaxis assay. Modified chemotaxis assay was used to study chemotaxis towards *Weissella* compared with *E. coli* OP50.

	30 min	60 min
95% Ethanol	69.3 ± 4.93^{e}	Not countable
Lactic acid	125.0 ± 4.00^{d}	Not countable
M9 buffer	8.7 ± 3.79^{c}	55.0 ± 3.61
E. coli OP50	39.0 ± 2^{a}	Not countable
W. koreensis	2.7 ± 0.58^{b}	Not countable
W. cibaria	2.3 ± 2.52^{b}	Not countable

The numbers represent the number of worms in the 30 mm-diameter-circle in the centre of plate including each bacterial lawn or test compound at 30 min and 60 min after feeding. Not countable means more than 300 worms (N2).
Different lowercase letters show the differences at $p < 0.05$ with Duncan's method.

Table S3. Oligonucleotide primers used in this study. Primers against age-related gene were designed using NCBI and Primer 3 software.

Target (accession #)	Primer	Sequence	Product size (bp)
act-2 (NM_073417)	Forward	5'-CCCACTCAATCCAAAGGCTA-3'	168
	Reverse	5'-GGGACTGTGTGGGTAACACC-3'	
daf-2 (NM_065249)	Forward	5'-GCCCGAATGTTGTGAAAACT-3'	185
	Reverse	5'-CCAGTGCTTCTGAATCGTCA-3'	
daf-16 (AF032112)	Forward	5'-TCCTCATTCACTCCCGATTC-3'	175
	Reverse	5'-CCGGTGTATTCATGAACGTG-3'	
sod-3 (NM_078363)	Forward	5'-GGATGGTGGAGAACCTTCAA-3'	185
	Reverse	5'-AAGGATCCTGGTTTGCACAG-3'	
skn-1 (NM_171345)	Forward	5'-TCAGGACGTCAACAGCAGAC-3'	164
	Reverse	5'-CGTGGAGATTCCGAAGAGAG-3'	
hif-1 (AF364604)	Forward	5'-AGGGGTTACCACCAGAGCTT-3'	217
	Reverse	5'-TTTTGCTGTTGCTGTCCTTG-3'	
jkk-1 (AB024086)	Forward	5'-CATCGCCATCCAGAAGAGAT-3'	178
	Reverse	5'-ACCGTCGCAGATTCGACTAC-3'	
jnk-1 (AB024085)	Forward	5'-AGAAGCGTGGAAGAGGATCA-3'	159
	Reverse	5'-ATCCAAAGAGACAGCGTCGT-3'	
aak-2 (NM_001029697)	Forward	5'-TGCTTCACCATATGCTCTGC-3'	219
	Reverse	5'-GTGGATCATCTCCCAGCAAT-3'	
ser-7 (NM_171637)	Forward	5'-CTCGAGGCTTTTCAAGTTGG-3'	232
	Reverse	5'-CCACAGTGCTAGCAGATCCA-3'	
daf-1 (NM_001027988)	Forward	5'-CCTCCTGGAATCATGCCTAA-3'	183
	Reverse	5'-CGCTTCACCTCGGTAGTCTC-3'	

age-1 (U56101)	Forward	5’-GGCTGCCAAGCTGAGTTATC-3’	122
	Reverse	5’-GCAGAGATTTCGGAGTTTCG-3’	
akt-1 (NM_001028475)	Forward	5’-GGACGTTGTGTTGATTGGTG-3’	167
	Reverse	5’-CCGGTGAGCAAAGTTCTAGC-3’	
akt-2 (AF072381)	Forward	5’-TCGACGAACCAGAGGAAGTT-3’	160
	Reverse	5’-GGTGAACGCAAGCATACAGA-3’	
sir 2.1 (NM_001268556)	Forward	5’-TGGCTGACGATTCGATGGAT-3’	179
	Reverse	5’-ATGAGCAGAAATCGCGACAC-3’	
hsf-1 (AY559748)	Forward	5’-AGCAGCACGTCGTTATGTTC-3’	181
	Reverse	5’-TACTGGAAGCTTGTCGTCGT-3’	
isp-1 (NM_068960)	Forward	5’-CGTACCAAGGCTGAGATTGC-3’	199
	Reverse	5’-CGTCGTAGTGAGATCCGTGA-3’	
vhl-1 (NM_077488)	Forward	5’-GTGGACGTGTTTTGGCTGAA-3’	186
	Reverse	5’-ATTCATTCGAGGAGCTGGCT-3’	
bli-3 (NM_058285)	Forward	5’-TTCAGCGTTCTTGGAGACCT-3’	188
	Reverse	5’-TGCAAAATGACAAGGCTCCC-3’	
duox-2 (NM_058283)	Forward	5’-GCATCCACGCTTGTTGATCT-3’	175
	Reverse	5’-TGTGTGTCTCCAGGATTCCC-3’	
